# A long-term experience of day-case pelvic osteotomy for developmental dysplasia of the hip

**DOI:** 10.1007/s11845-025-03963-y

**Published:** 2025-05-09

**Authors:** Dave M. Moore, Catherine Howells, Olga Gallagher, David P. Moore, Pat O’Toole

**Affiliations:** Department of Trauma and Orthopaedic Surgery, Childrens Health Ireland, Crumlin, Cooley Rd, Crumlin, Dublin, D12 N512 Ireland

**Keywords:** Day-case, DDH, Developmental dysplasia of the hip, Outpatient surgery, Pelvic osteotomy

## Abstract

**Objective:**

Developmental hip dysplasia has an incidence of 6.73 per 1000 live births and leads to a significant number of orthopaedic referrals annually. This high demand has encouraged the drive to optimize the efficiency of service provision in the paediatric orthopaedic setting. Here we describe our long-term experience with a novel day-case pelvic osteotomy initiative. We also describe any potential complications one can expect when performing day-case pelvic osteotomies.

**Methods:**

This was a non-randomized prospective cohort study conducted to compare conventional in-patient pelvic osteotomies with day-case osteotomies performed between January 2017 and November 2023. All surgeries took place at an urban tertiary national referral centre by four paediatric orthopaedic surgeons with a specialist interest in DDH.

**Results:**

164 Salter and Pemberton osteotomies were performed of which 115 met the day-case criteria. Based on the HSE ‘Specialty Costing Report’ and ‘Annual Report and Financial Statements’, the total discharge cost for patients undergoing an in-patient osteotomy was €6619 in contrast to €2670 per day-case patient. For the 110 day-cases, the cost to treat amounted to €293,700; hence, there was a total saving of €434,390 made by the hospital for the 110 day-cases performed. This amounts to €3949 saved for every day-case.

**Conclusion:**

Review at 7 years has demonstrated that day-case pelvic osteotomy surgery for DDH remains a safe and cost-effective initiative that significantly reduces the demand on in-patient hospital bed resources.

## Introduction

Developmental dysplasia of the hip (DDH) refers to a spectrum of pelvic and hip abnormalities ranging from instability in 1 in 100 live births, subluxation and dislocation in 1 in 1000 live births [1–4]. In Ireland, DDH has an incidence of 6.73 per 1000 live births [5] and leads to a significant number of referrals to orthopaedic services annually. This high demand has encouraged the drive to optimize the efficiency of service provision in the paediatric orthopaedic setting.

Around the world, out-patient operative activity is becoming more widespread [6–12]. We have seen it has successful implementation in the adult trauma and elective setting [6–12]; however, uptake in paediatric orthopaedics has not been as prevalent. We are now living in the era of integrated care systems, with the aim of transferring our paediatric care back to the community [12] and reducing the burden on tertiary referral centres in the UK and Ireland. With current waiting list times in Ireland still exceeding the acceptable time frame [13], it has never been more imperative to optimize efficiencies to ensure a higher productivity and throughput in the elective paediatric orthopaedic setting.

To date, our pilot study on the benefits of performing pelvic osteotomies as day-case procedures in a paediatric population is the only publication of its kind in the literature [14]. Here we report our long-term experience with a novel day-case pelvic osteotomy initiative in a national tertiary referral centre. We describe the clinical and administrative support structures required to safely introduce this service. We also describe any potential complications one can expect when performing day-case pelvic osteotomies.

## Methods

This was a non-randomized prospective cohort study conducted to compare conventional in-patient pelvic osteotomies with day-case osteotomies performed over a 7-year period between 1 st January 2017 and the 1 st of January 2024. All surgeries took place at an urban tertiary national referral centre and were performed by four paediatric orthopaedic surgeons with a specialist interest in DDH. Patients with a diagnosis of DDH, ASA status I or II, undergoing either a Salter or Pemberton osteotomy and living within 50 kms of the hospital were enrolled in the study. Exclusion criteria were an inability to comply with a predetermined analgesic protocol or the presence of obstructive sleep apnoea as determined at anaesthetic pre-assessment review.

### Surgical technique

Decision to treat DDH operatively was based on a raised acetabular index and it is the unit’s policy not to operate before 18 months. We often wait until patients are between 2- and 3-years old to allow cases a chance to resolve with conservative measures. Salter and Pemberton osteotomies were performed using well-described and standard surgical techniques.

Information was collected prospectively by DDH clinical nurse specialists and recorded in our local DDH database. Clinical parameters recorded included age, gender, DDH risk factors, unscheduled post-operative attendances to the emergency department (ED) or out-patient department and readmission to hospital for any reason. The primary parameters assessed included clinical and radiographic outcomes. The financial implications of this novel process and the reduction in in-patient resources were included in the secondary outcomes of the study.

### Pre-operative preparation

For all cases included in the day-case group, a strict protocol involving anaesthetic, surgical and nursing staff was implemented from the time of admission through to discharge as follows:

All parents of patients eligible to undergo day-case surgery were informed of the need to assess pain and administer regular analgesia at home post-operatively. An information leaflet with all relevant information was provided along with a prescription for oral morphine to ensure patients had necessary analgesia on discharge. Any concerns or questions parents had around out of hospital use of opiates including signs of tolerance or toxicity was addressed by the Orthopaedic and Anaesthetic practitioners.

### Intra-operative analgesia

All patients had a single shot caudal epidural or fascia iliaca block with magnesium sulphate as an additive. If a block was not possible or failed, the alternative regimen of morphine 0.2 mg/kg intramuscularly and lidocaine 2 mg/kg IV was used. Analgesia consisting of diclofenac (per rectum), dexamethasone and clonidine were administered intraoperatively. All patients were placed in a hip abduction orthosis directly after their osteotomy for comfort.

### Post-operative analgesia

On the first post-operative day, all parents received a phone call from the DDH clinical nurse specialist enquiring how the patient was, if pain was controlled and to ensure there were no parental concerns. All patients had follow-up clinic visits at 6 weeks. Standardized post-operative analgesics were used in all cases with regular paracetamol (15 mg/kg 6-hourly PO) and ibuprofen (7.5–10 mg/kg PO 8 hourly) being prescribed on discharge and oral weight based morphine once a day on post-operative days one, two and three and then stopped. Parents are advised that they have eight doses of morphine to be used if needed and that a three day course of opioids to be administered as needed following surgery is appropriate for effective pain control [15].

In July 2022, the ‘Health Service Executive (HSE) Annual Report and Financial Statements’ was published [16]. This contained the specific total costs of performing paediatric pelvic osteotomies as day-case procedures and as standard in-patient procedures. Using this information, we extrapolated the ‘total discharge cost’ (i.e. the complete cost to the hospital) for both day-case and conventional in-patient pelvic osteotomies.

The Strengthening the Reporting of Observational Studies in Epidemiology (STROBE) checklist was adhered to throughout the study [17]. Simple descriptive statistics were used to describe our findings.

## Results

One hundred sixty-four Salter and Pemberton osteotomies were performed between 1 st January 2017 and 30 th November 2023. Of these, 115 hips met the day-case criteria with a mean follow-up of 5.2 years. The remaining 49 patients were treated as inpatients with a length of stay of 2 days. Five patients in the day-case group were kept in overnight following surgery and removed from the day-case cohort. Three of these were due to inadequate pain control or parental concern, and two were for post-operative vomiting. Taking these into account, 110 patients completed their operative journey as day-cases and there was a total reduction of 220 in-patient bed days reported over the 83-month period.

**Fig. 1:**
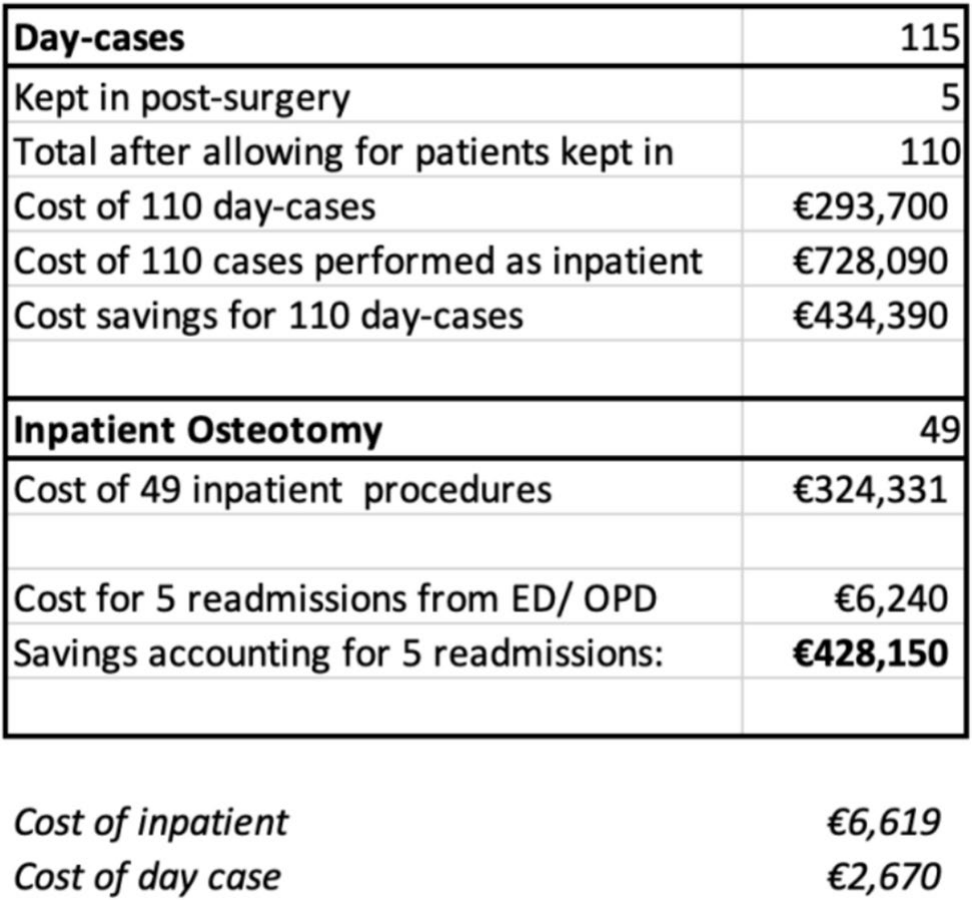
Summary of total discharge costs

Based on the HSE ‘Specialty Costing Report’ and ‘Annual Report and Financial Statements’ [16], the ‘total discharge cost’ for patients undergoing an in-patient osteotomy was €6619 per patient. In contrast, the total discharge cost of performing the procedure as a day case was reported at €2670 per patient. For the 110 day-case patients, the total cost to treat amounted to €293,700. Calculating the difference, there was a total saving of €434,390 made by the hospital for the 110 day-cases performed. This amounts to €3949 saved for every day-case (see Fig. 1 for an overview of the costs).

Following day-case discharge, 22 patients (20%) had unscheduled presentations to the emergency department or outpatient clinic in the days following surgery of which five kept in overnight for analgesia optimisation and discharged the next day (four of these were due to inadequate analgesic control secondary to non-compliance and one was for gastritis).

The total cost of emergency department attendance and one overnight admission was €1248. Therefore, adjusting for these five re-admissions in the day-case group, the overall saving to our institution with the introduction of this novel day-case model of care was €428,150. The other 88 day-case patients reported adequate pain control with no concerns warranting unscheduled attendance.

### Complications

**Fig. 2:**
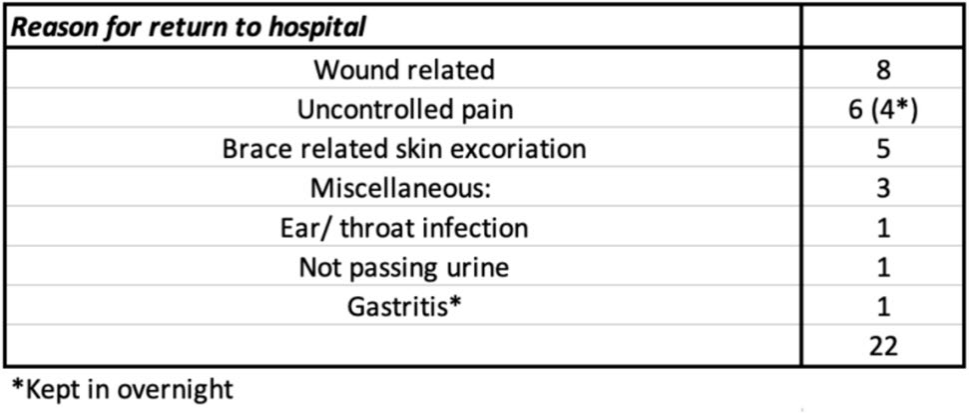
Cause for re-attendance to the hospital

There were no recorded cases of hardware failure, osteotomy displacement or reoperation in the day-case cohort (see Fig. 2 for a summary of reasons for re-attendance).

Clinical and radiographic outcomes were the same in both groups with no complications reported on follow-up. Of note, eight (7.2%) patients that re-attended the emergency department had wound ooze or suspicion of superficial wound infection, but all were discharged from the emergency department with antibiotics and had successful resolution of symptoms at subsequent out-patient review. We do not consider this as a true complication but have mentioned it in order to ensure transparency and to inform the reader that wound concerns should be expected.

## Discussion

In 2020, we originally described the first application of day-case orthopaedic paediatric osteotomies in the literature [14]. Over a longer period of time and with more experience, we have continued to show that day-cases are equally safe, radiographically equivalent and financially preferable to the traditional in-patient model of care. Our original publication had four re-attendances to the emergency department which was 11.4% of the day-case cohort. The 20% re-attendance rate in this review is significantly higher and one that cannot be overlooked. Eight of the 22 patients that returned were due to concern around wound erythema or discharge which prompted us to review our practises around brace application and wound closure. It provides valuable information when counselling parents around what they can expect in their child’s peri-operative journey. We now advise them that whilst most children go home on the day of surgery without issue, two out of ten will return due to various concerns and will likely not require intervention following review.

Out-patient operative activity continues to evolve and its application is becoming more widely adopted. Day-case surgical procedures have been introduced with notable success in other areas such as trauma, knee, hip and shoulder arthroplasty [6, 7, 9–12, 14]. Whilst it has been shown that day-case surgery is a welcome development among adult patients [7], it remains a rarity in the paediatric orthopaedic setting.

We do acknowledge the challenges in order to succeed in establishing an out-patient programme, including the presence of a clinical nurse specialist, careful patient selection, pre-operative patient education and specialized protocols [18]. Proper patient selection during preoperative assessment, robust inclusion/exclusion criteria, education and a well-defined clinical pathway are all essential for success. Prevention of postoperative nausea and vomiting should not be overlooked as this was the reason for two patients remaining in hospital in our study. With the current health economic climate we are experiencing in Britain and Ireland, the expense of keeping a patient in overnight for nausea and vomiting should not be ignored.

Our reattendance rates are in line with those reported by Fishley et al. who found that wound concerns were the predominant reason for patients to return to hospital following day-case surgery [6]. Similarly to our experience, no patients required a revision procedure or developed metalwork infection [6]. They concluded that day-case surgery can be delivered safely with no additional resources. Leroux et al. concluded that 30-day adverse event and readmission rates for total shoulder arthroplasty were not significantly different between in-patients and out-patients [11]. Although there are concerns around early readmission rates in some studies for out-patient surgery, our findings corroborate the theory that, for selected surgical candidates undergoing a suitable surgical procedure, day-case surgery is safe. We report no negative long-term outcomes in the day-case cohort compared to the in-patient group in this study. Our re-review rates are significantly higher than those reported for the elective adult day-case total hip and knee arthroplasty (THA/TKA) population. Emergency department re-attendance for adults following day-case THA/TKA are reportedly as low as 1.9% [19] with readmissions being 3.9% [20], both significantly lower than what we have experienced. We acknowledge that in many ways our paediatric cohort cannot be compared to adults as a significant proportion of emergency department visits were for parental concern. Paediatric patients are often times not able to communicate their pain effectively therefore there is likely a lower threshold to re-attend the emergency department in this population.

The benefits to both the child and parents are significant, by decreasing the psychological and emotional distress associated with an overnight hospital stay whilst also improving hospital efficiency, cost effectiveness and increasing inpatient bed availability. Some patients may have comorbidities which render them poor candidates for day-case procedures and general anaesthesia. Proper patient selection, education and a well-defined clinical pathway are all key for successful day-case surgery [14]. Shaw et al. concluded that pre-operative admission status had no effect on peri-operative complications, readmission or unplanned operation rates and deemed out-patient spinal lengthening to be safe [21]. Similarly, Stull et al. compared the outcomes and cost-effectiveness of out-patient versus in-patient ankle fracture surgery on a selected cohort [8]. Our findings also confirm the cost-effectiveness of day-case surgery as there was a significantly decreased cost for the out-patient group compared to in-patient cases. Stull also noted that if implemented on a national level in the USA, out-patient surgery for ankle fractures would lead to a $376,705,438 saving for the treatment of that injury [8].

Day-case procedures are not without risk however. In a retrospective review to determine complication rates associated with out-patient total knee arthroplasty compared with inpatients, Arshi et al. found that day cases were more likely to undergo revision, explanation of the prosthesis and manipulation under anaesthesia within 1 year [22]. Higher incidences of post-operative deep vein thrombosis were also noted in this article. These findings were not reproduced in our experience or that of Fishley et al. [6], as none of the patients in our cohorts required revision surgery or removal of metalwork at final follow-up. Day-case surgery may only be suitable for certain procedures. We recognize that prior to implementation of a day-case surgical programme, the inclusion and exclusion criteria must be rigid and safe regarding potentially complicated patients. We acknowledge the importance of the multi-disciplinary approach to the safe introduction of an elective day-case paediatric orthopaedic initiative. Further larger-scale paediatric specific studies are necessary to demonstrate the safety of day-case surgery and in order to corroborate our findings.

### Limitations

Limitations to our study are that patient samples were not randomized, however, on comparison, there were no differences in the demographics or risk factors for the two groups. Certain patients underwent conventional in-patient osteotomy as this was the surgeon’s preference. We are still in the process of an institutional move towards day-case osteotomy for all patients.

## Conclusion

Review at 7-years has demonstrated that day-case pelvic osteotomy surgery for DDH remains a safe and cost-effective initiative that significantly reduces the demand on in-patient hospital bed resources but up to 20% of patients may attend the hospital for re-review prior to planned clinic date. Savings of €3949 per day case have significant future financial implications for all treating institutions. This novel out-patient model of care is also clinically equivocal to the traditional in-patient model of care for DDH pelvic osteotomies.
